# Diffusion-Weighted MRI in Perianal Abscess: Role and Comparison With Contrast-Enhanced MRI

**DOI:** 10.7759/cureus.59035

**Published:** 2024-04-25

**Authors:** Pooja Aggarwal, Rajesh Malik, Radha Sarawagi, Aman Kumar, Jitendra Sharma

**Affiliations:** 1 Radiology, All India Institute of Medical Sciences, Bhopal, IND

**Keywords:** t2 short tau inversion recovery, perianal fistula, perianal abscess, contrast enhanced mri, diffusion weighted imaging

## Abstract

Background: Perianal abscess is a clinical infective and/or inflammatory collection in the perianal region, one entity of a large group of anal and perianal disorders. Perianal abscesses are often seen as a complication of grade 2 and grade 4 perianal fistulas from St. James’s University Hospital classification. Several imaging modalities have been tried in the past for adequate assessment of perianal abscess with contrast-enhanced magnetic resonance imaging (CE-MRI) providing the most accurate results. Diffusion-weighted imaging (DWI) is an emerging sequence that can provide comparable results to CE-MRI in diagnosing and characterizing perianal abscess. The main objective of this study is to assess the role of DWI in adequate identification and assessment of perianal abscess and compare the final results with contrast-enhanced images.

Methods: Twenty patients with complicated perianal fistula with clinically suspected perianal abscess were evaluated with DWI and CE-MRI. This study was a comparative cross-sectional study conducted in the Department of Radiodiagnosis and Imaging, All India Institute of Medical Sciences, Bhopal, India. Chi-square test was done to find the association between categorical variables. Kappa test was used to find the agreement between two different tests. Receiver operating characteristics (ROC) analysis was done to estimate the area under the curve in predicting the outcome. Sensitivity, specificity, positive predictive value, negative predictive value and accuracy were used to measure the validity of the tests.

Results: DWI is a very sensitive MRI sequence and is equivalent to CE-MRI to detect the location and analyzing the loco-regional extent of abscess in complicated perianal fistula cases. DWI is also very sensitive and superior to T2 short tau inversion recovery (STIR) in differentiating perianal abscess from perianal inflammation without abscess.

Conclusion: DWI can be used as an alternative to post-contrast fat-suppressed MRI in precisely defining the location and extent of anal and perianal abscesses and disease activity in complicated fistula cases.

## Introduction

Anal and perianal abscesses are infective/inflammatory collections filled with pus, inflammatory cells and/or granulation tissue. They are often seen in the setting of complicated perianal fistula but can also be seen as the precipitating cause of perianal fistula. Perianal abscess is a part of a wide group of anorectal abscesses, and is the most common type of it. They are mainly confined to the perianal region but if left untreated, can extend to involve the continuations of the perianal space. In the worst and most troublesome cases, these can also lead to systemic infections [1,2].

Common causes of perianal abscess include trauma, anal sex, diabetes mellitus, corticosteroid medications, immunosuppressive state, anal fissures and inflammatory bowel disease such as Crohn's disease [1,3]. The pathophysiology of perianal abscess involves occlusion of the drainage system of anal glands and subsequent infection of these anal glands by various aerobic and anaerobic organisms [4] which in severe cases can form an abscess and if not adequately treated or surgically drained, these can spread to the surrounding communicating spaces of least resistance including peri-rectal, intersphincteric, ischiorectal or supralevator space [5,6].

Patients commonly present with redness and swelling in the anal region often associated with throbbing pain, fever, tenderness, constipation and perianal discharge (in cases of fistulous tract formation). Males are more commonly affected as compared to females [7]. Majority of the perianal abscesses are formed as a complication of pre-existing perianal fistula, commonly seen in grade 2 and 4 of St. James’s University Hospital classification, which uses magnetic resonance imaging (MRI) interpretation to classify perianal fistula [8].

Early diagnosis of the condition is essential for timely intervention. Usually, clinical examination is sufficient to diagnose the presence of abscess as the cause of the patient’s symptoms. However, to accurately evaluate the location, extent and possible associated complications, further radiological investigation is often needed. Various imaging modalities have been tried for decades including CT pelvis and CT/Xray-fistulography, but did not prove to be of much use. Anorectal ultrasound is a useful modality for identification and characterization of perianal abscess; however, its use is often limited owing to extreme pain and tenderness suffered by the patient due to underlying abscess. Transcutaneous perineal ultrasonography using high-frequency linear transducer can be used to identify the location of abscess, measure its approximate volume and grossly assess its loco-regional extent, however, detailed analysis can still not be done [9]. Thus, it requires further imaging especially in deep and extensive cases. Here comes the role of MRI which is now used as the modality of choice for perianal abscesses with or without active perianal discharge as it demonstrates excellent soft tissue contrast and thus extensively evaluates even the finer details and extensions of abscess which helps in appropriate surgical planning, thereby reducing the overall recurrence rate.

Contrast-enhanced MRI (CE-MRI) is currently the gold standard for perianal disease with almost 100% accuracy when compared with post-surgical outcomes. Diffusion-weighted imaging (DWI) is another technique that has high detection rate for abscesses in any part of the body [10]. It is based on Brownian motion i.e. the changes in movement of water molecules that happen when water molecules interact with cell membranes. The level of diffusion of water molecules is inversely proportional to the cellularity of tissue. Thus, high cellular environment, as seen in inflammation and neoplastic conditions, restricted diffusion of water molecules is seen which is interpreted as diffusion restriction on DWI [11].

## Materials and methods

Our study is an observational study of comparative cross-sectional design, conducted at All India Institute of Medical Sciences Bhopal, a tertiary care hospital, after approval from the Institutional Human Ethics Committee (IHEC) (approval number: IHECPGRMD035). Patients above 18 years of age with grade 2 and 4 perianal fistula and suspicion of perianal abscess were included in our study after obtaining written informed consent. Patients who had a history of fistula surgery, history of claustrophobia, non-consenting patients, contraindications for MRI such as metallic implants or contraindications to contrast agents and non-interpretable images were excluded from the study. Patients from other types of fistulas were also excluded.

MRI acquisition

MRI for all the refereed patients were performed at All India Institute of Medical Sciences, Bhopal, India, using a 1.5T superconducting system using an eight-channel phased-array body multicoil. A minimum fasting period of six hours was given to all the patients. After selecting proper localiser and field of vision (FOV), an initial sagittal T2 fast spin echo (FSE) sequence was taken in all the patients for correct orientation and axis of anal canal, and then tilting the anal canal by ~45^0^ to obtain oblique axial, oblique coronal and sagittal images. Plain scans were taken using 3-5 mm slice thickness followed by injection of gadolinium (at concentration of 0.1 mL/kg through 20G intravenous catheter) showing contrast enhancement on T1 weighted images.

MRI protocol used in our study included following sequences: axial T1, high-resolution T2 short tau inversion recovery (STIR) in axial and coronal, high-resolution T2 in all three planes, axial DWI, pre-contrast fat suppressed (FS) T1 in axial and post-contrast fat suppressed T1 in all three planes (Table 1).

**Table 1:** MRI protocol for perianal abscess. DWI: diffusion weighted imaging, STIR: short tau inversion recovery, FS: fat suppressed, TR: repetition time, TE: echo time, FOV: field of view

MRI sequence	Plane	TR/TE (ms/ms)	Matrix size (mm^2^)	FOV (mm^2^)
DWI	Axial	6200/61	120x160	260x260
T2	Sagittal	4940/93	269x384	200x200
T2	STIR Coronal	5250/54	269x384	316x316
T2	STIR Axial	5170/34	253x320	200x200
T2	Coronal	4590/83	269x384	316x316
T2	Axial	3550/80	269x384	200x200
T1	Sagittal	1020/12	269x384	200x200
T1	Axial	887/14	224x320	200x200
T1 FS Post contrast	Sagittal	1200/9.9	240x320	230x230
T1 FS Post contrast	Coronal	1310/11	205x256	230x230
T1 FS Post contrast	Axial	1150/14	203x320	199x220

MRI interpretation

The perianal fistulas were evaluated using all the obtained sequences as mentioned above. The data was recorded for the results of DWI, T2 STIR and post-contrast fat-suppressed T1W sequences in interpretation of perianal abscesses. The radiologist was provided with clinical details of the patients and was blinded to MRI findings. To avoid recall bias, analysis of all the MRI scans was done in two sessions at a three-week interval in which DWI and T2 STIR images were analysed in the first session and post-contrast fat suppressed T1 images were analysed in the second session. A dichotomous scale was used to record the findings wherein 0 was interpreted as “not visualized’ and 1 as ‘visualized’.

Contrast scans were considered as the reference gold standard and diagnostic accuracy of DWI was compared. DWI images with a b value of 1000 s/mm^2^ were used. Diffusion restriction was considered when DWI displayed a hyperintense signal with a corresponding drop in apparent diffusion coefficient (ADC).

Statistical analysis

Data was entered in MS Excel (Microsoft, Redmond, WA, USA) and analysis was done using SPSS version 21.0 (IBM Corp., Armonk, NY, USA). Data was recorded as percentages for categorical variables and as mean and standard deviation for continuous variables. To find the association between categorical variables, Chi-square test was used and to estimate the agreement between two different tests, Kappa test was done and outcome was measured. Receiver operating characteristics (ROC) analysis was done to estimate the area under the curve in predicting the outcome. Sensitivity, specificity, positive predictive value (PPV), negative predictive value (NPV) and accuracy were used to measure the validity of tests. P value of <0.05 was considered significant.

## Results

**Figure 1:**
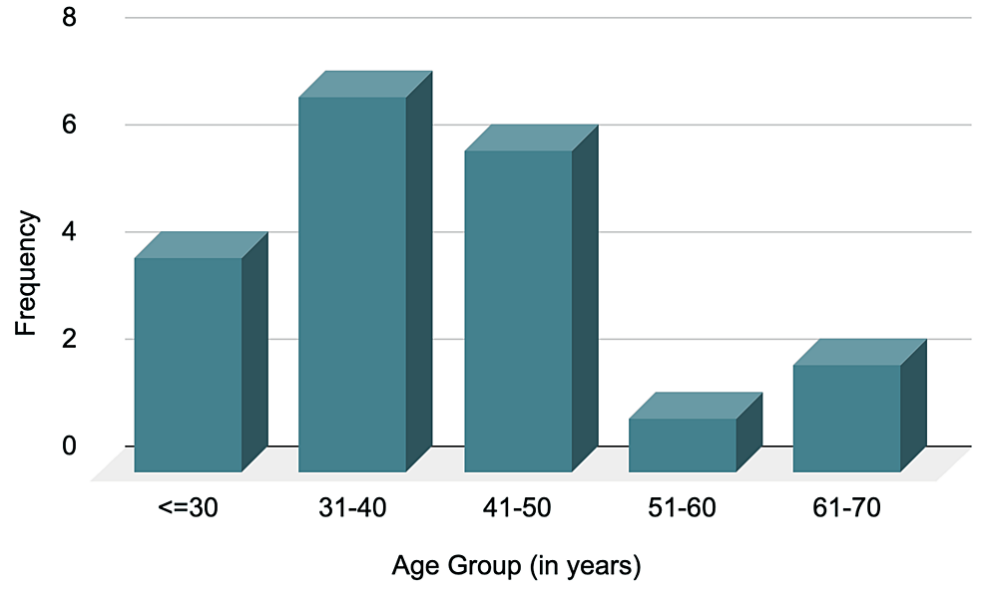
Bar chart depicting the age distribution of the patients.

**Figure 2:**
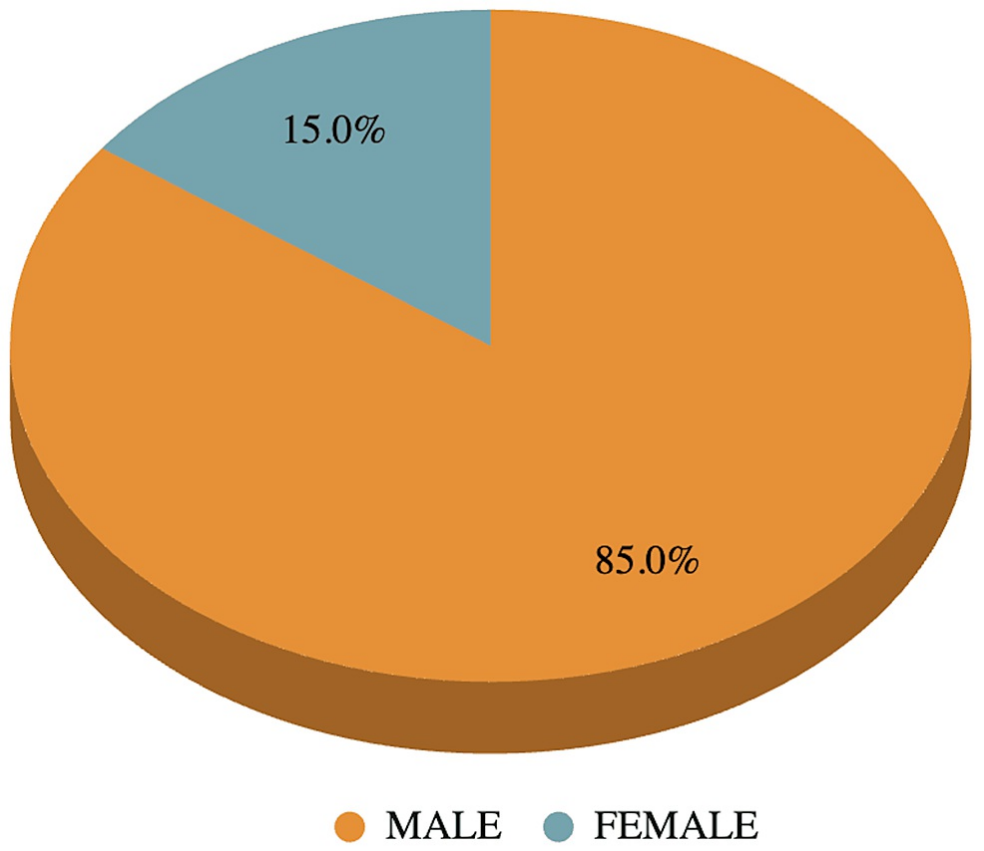
Pie chart depicting the gender distribution.

On demographic analysis, 20 patients with grade 2 and grade 4 perianal fistula complicated with perianal abscess met the inclusion criteria and are thus included in our study. The ages of the patients ranged from 23 to 70 years, with the mean age and standard deviation being 40.0 ± 12.1 years with majority of them in 31-40 years range (Figure 1). Out of 20 patients, 17 (85%) patients were males and three (15%) were females (Figure 2).

**Figure 3:**
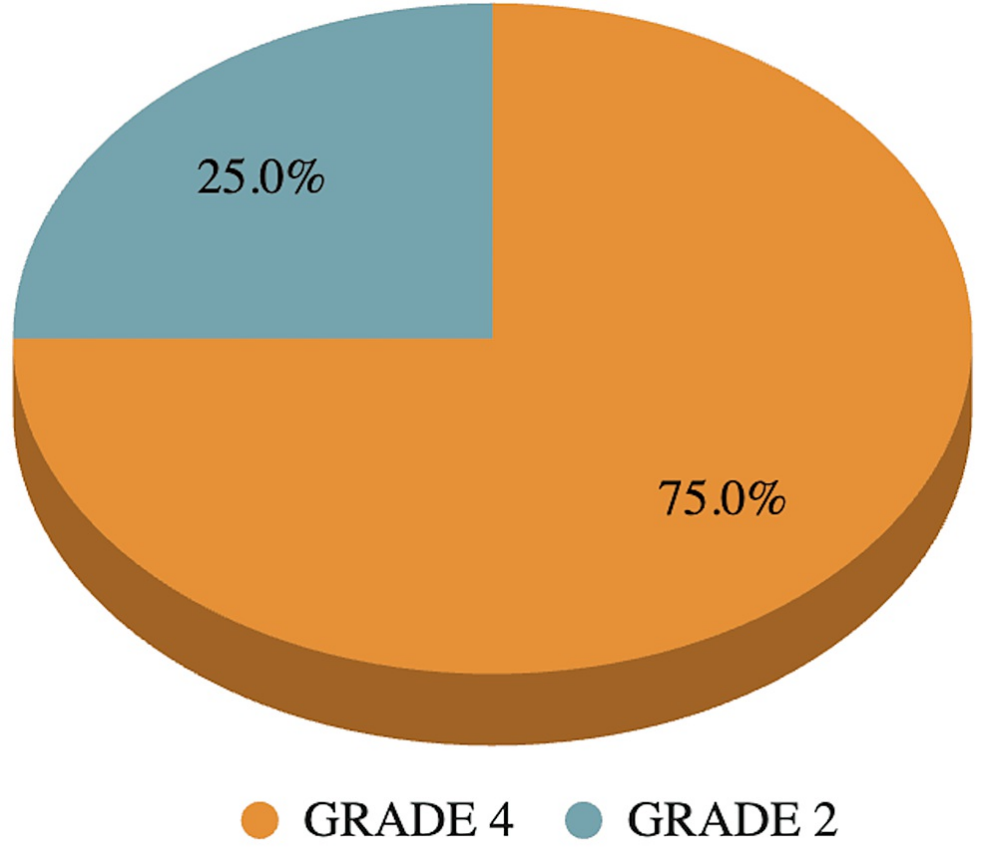
Pie chart depicting distribution of fistulas according to St. James’s University Hospital Classification.

Of all these patients, five (25.0%) had grade 2 perianal fistula and the rest (15, 75.0%) had grade 4 fistula according to the St. James’s University Hospital classification (Figure 3). Out of a total of 20 cases, 16 cases showed perianal abscess whereas four patients did not show abscess on post-contrast T1 FS imaging.

Of these 16 abscesses identified on post-contrast fat-suppressed T1 images, 13 abscesses were identified on DWI and 12 cases were identified on T2 STIR (Table 2). 

**Table 2:** Frequency of detection of perianal abscess by DWI and T2 STIR in comparison to CE-MRI. DWI: diffusion weighted imaging; STIR: short tau inversion recovery; CE: contrast enhanced

ABSCESS	FREQUENCY	PERCENTAGE
DWI	13	81.2
T2 STIR	12	75.0
CE-MRI	16	100.0

DWI had an accuracy of 85.00%, sensitivity of 81.25% (95% CI: 54.35% to 95.95%), and specificity of 100% (95% CI: 39.76% to 100.00%) (Table 3). Almost perfect agreement was found between DWI and post-contrast fat-suppressed T1 WI (P < 0.001, kappa: 0.874) (Table 4).

**Table 3:** Diagnostic performance of DWI in detection of perianal abscess. DWI: diffusion weighted imaging; CI: confidence interval

DWI	Value	95% CI
Sensitivity	81.25%	54.35% to 95.95%
Specificity	100.00%	39.76% to 100.00%
Positive Predictive Value	100.00%	75.29% to 100.00%
Negative Predictive Value	57.14%	32.47% to 78.71%
Accuracy	85.00%	62.11% to 96.79%

**Table 4:** Agreement between DWI and CE-MRI in detecting perianal abscess. DWI: diffusion weighted imaging; CE: contrast enhanced

DWI	CE-MRI		TOTAL	CHI-SQUARE TEST, P VALUE
	YES	NO		
YES	13	0	13	P<0.001, KAPPA: 0.874
	81.3%	0.0%	65.0%	
NO	3	4	7	
	18.8%	100.0%	35.0%	
TOTAL	16	4	20	
	100.0%	100.0%	100.0%	

Twelve cases were identified on T2 STIR with an accuracy of 80.00%, sensitivity of 75.00% (95% CI: 47.62% to 92.73%), and specificity of 100% (95% CI: 39.76% to 100.00%) (Table 5). Almost perfect agreement was found between T2 STIR and post-contrast fat-suppressed T1 WI (P < 0.001, kappa: 0.828) (Table 6).

**Table 5:** Diagnostic performance of T2 STIR in detection of perianal abscess. STIR: short tau inversion recovery; CI: confidence interval

T2 STIR	Value	95% CI
Sensitivity	75.00%	47.62% to 92.73%
Specificity	100.00%	39.76% to 100.00%
Positive Predictive Value	100.00%	73.54% to 100.00%
Negative Predictive Value	50.00%	29.79% to 70.03%
Accuracy	80.00%	56.34% to 94.27%

**Table 6:** Agreement between T2 STIR and CE-MRI in detecting perianal abscess. STIR: short tau inversion recovery; CE: contrast enhanced

T2 STIR	CE-MRI		TOTAL	CHI-SQUARE TEST, P VALUE
	YES	NO		
YES	12	0	12	P<0.001, KAPPA: 0.828
	75.0%	0.0%	60.0%	
NO	4	4	8	
	25.0%	100.0%	40.0%	
TOTAL	16	4	20	
	100.0%	100.0%	100.0%	

**Figure 4:**
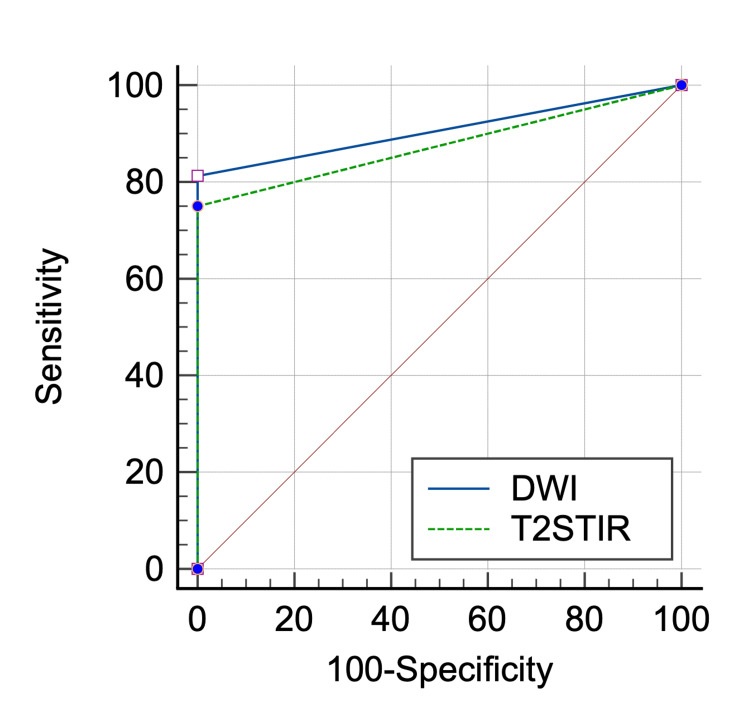
Receiver-operating characteristic (ROC) analysis of 2 parameters (DWI and T2 STIR respectively) for the diagnosis of perianal abscess with CE-MRI as gold standard. DWI: diffusion weighted imaging; STIR: short tau inversion recovery; CE: contrast enhanced

Table 7 and Figure 4 show ROC analysis of DWI to estimate the area under the curve in predicting the outcome and comparing the results with ROC analysis of T2 STIR.

**Table 7:** Receiver-operating characteristic (ROC) analysis of DWI and T2 STIR for the diagnosis of perianal abscess with CE-MRI as gold standard. DWI: diffusion-weighted imaging; STIR: short tau inversion recovery; AUC: area under the curve; SE: standard error; CI: confidence interval; CE: contrast enhanced

PERIANAL ABSCESS	AUC	SE	95% CI
DWI	0.906	0.0504	0.807 to 1.000
T2STIR	0.875	0.0559	0.765 to 0.985

## Discussion

Abscess formation in the perianal region can be caused by a variety of reasons. In our study, we have taken perianal fistula cases complicated with abscess. Perianal abscess is not an uncommon complication of perianal fistula, commonly seen in grade 2 and grade 4 from St James’s University Hospital classification. Patients with perianal fistulas complicated with abscess often present with red, painful, tender swelling in the perianal region which can further get complicated and extend into continuations of perianal space. Our study included majority of grade 4 perianal cases (75%) and few grade 2 fistula cases (25%). Out of total 20 cases, only 16 patients were found to have abscess formation in post contrast T1 FS images, which we have considered a gold standard in our study. Of these 16 cases, 12 cases (75%) were from grade 4 and four cases (25%) were from grade 2 perianal fistula.

Perianal abscess has been a troublesome condition for patients, affecting their daily routine activities and thus has been studied since many years for proper management. Many different imaging modalities have been tried in the past, out of which ultrasonography is the most convenient and readily available investigation. Trans-anal ultrasound is often difficult to tolerate by patients in an already existing painful condition, however transcutaneous perineal ultrasound can offer diagnostic advantage in superficially assessing location and loco-regional extent of collection specially in emergency cases [9,12,13].

Although ultrasound was able to offer its own benefits in emergency and mild cases, deep seated, widely extended and complicated conditions still needed more extensive detailing for better surgical intervention with the aim to reduce post treatment recurrence rates.

MRI is the latest and most promising modality for accurate and detailed exploration of abscess collection with detailed anatomy of associated perianal fistulas. MRI is currently the investigation of choice for uncomplicated and complicated perianal diseases. Standard MRI protocol used contrast enhanced images as the gold standard technique with maximum sensitivity when compared to post-surgical outcomes [14,15].

Diffusion weighted imaging has gained popularity in recent times owing to its superb soft tissue contrast in abscess collection differentiating it from surrounding normal soft tissue. The role of DWI in detecting perianal fistula relies on its ability to detect pus filled active fistulous tracts where it shows hyperintense signals with corresponding drop on ADC (referred as restricted diffusion) [16,17]. The inability of T2 STIR to differentiate between the hyperintense signal in the active fistulous tract from perianal inflammatory surrounding can be overcome by diffusion weighted imaging which shows restriction only in active fistula tract and only hyperintense signal on DWI with no signal drop on ADC (and hence no diffusion restriction). Similarly, DWI surpasses T2 STIR in differentiating abscess from perianal inflammation (without abscess) with contrast being the most sensitive and gold standard. While contrast is still the most sensitive sequence in diagnosing and assessing the characteristics of abscess in perianal fistula cases, DWI can be used to replace contrast injection with comparable accuracy. Same results we found in our study wherein we found DWI to be equivalent to contrast enhanced images and superior to T2 STIR in perianal abscess characterization and differentiating it from surrounding non-specific inflammation.

Few limitations of our study included not using post-surgical outcomes for more efficient comparison and secondly, we used 1000 s/mm² b value for DWI sequence and hence the final results may vary from higher b values. Another limitation of our study was small sample size.

**Figure 5:**
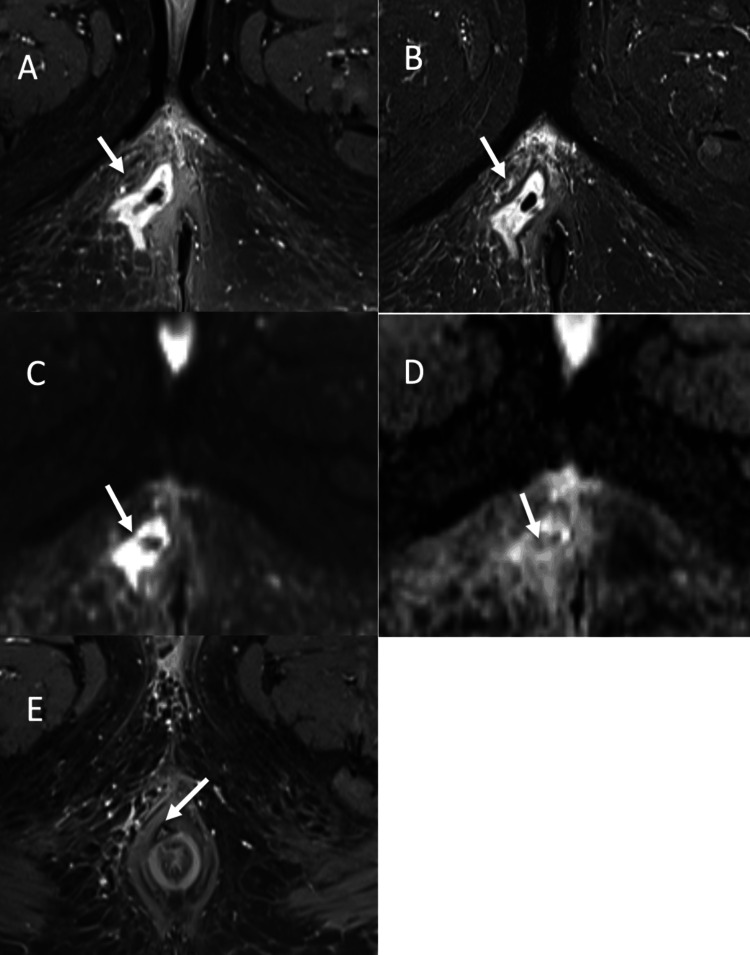
Perianal abscess in grade 4 trans-sphincteric fistula: (A-B) Axial post contrast fat suppressed T1-WI and axial STIR respectively, depicting a large heterogeneous collection with internal air foci in right perianal region showing peripheral enhancement on post contrast fat suppressed T1-WI (A) and hyperintense signal on T2 STIR (B). (C-D) Axial DWI and ADC respectively, depicting the collection showing hyperintense signal on DWI (C) with corresponding signal drop on ADC (D). (E) Axial post contrast fat supressed T1-WI showing a small trans-sphincteric fistula crossing the external sphincter at 11-o’clock position. T1-FS: T1 fat suppressed; STIR: short tau inversion recovery, DWI: diffusion weighted imaging; ADC: apparent diffusion coefficient

**Figure 6:**
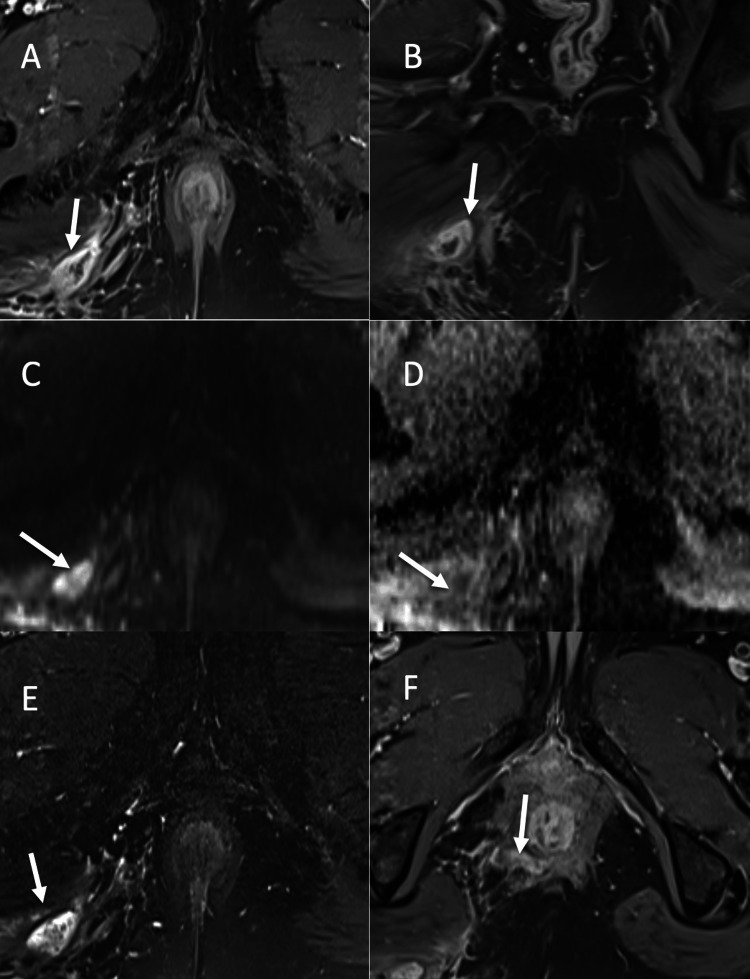
Perianal abscess and inflammation in grade 4 trans-sphincteric fistula: (A-B) Axial and coronal post contrast fat suppressed T1-WI respectively, showing heterogeneously and predominantly peripherally enhancing abscess collection in right perianal region. (C-E) Axial DWI, ADC and STIR respectively, depicting abscess showing hyperintense signal on DWI (C) with corresponding signal drop on ADC (D) and hyperintense signal on T2 STIR (E). Note the perianal inflammation showing hyperintense signal on T2 STIR with not restriction on DWI. (F) Axial post contrast fat suppressed T1-WI showing a curvilinear trans-sphincteric fistula coursing the external sphincter at 8-o’clock position. T1-WI: T1 weighted image; DWI: diffusion weighted imaging, ADC: apparent diffusion coefficient; STIR: short tau inversion recovery

**Figure 7:**
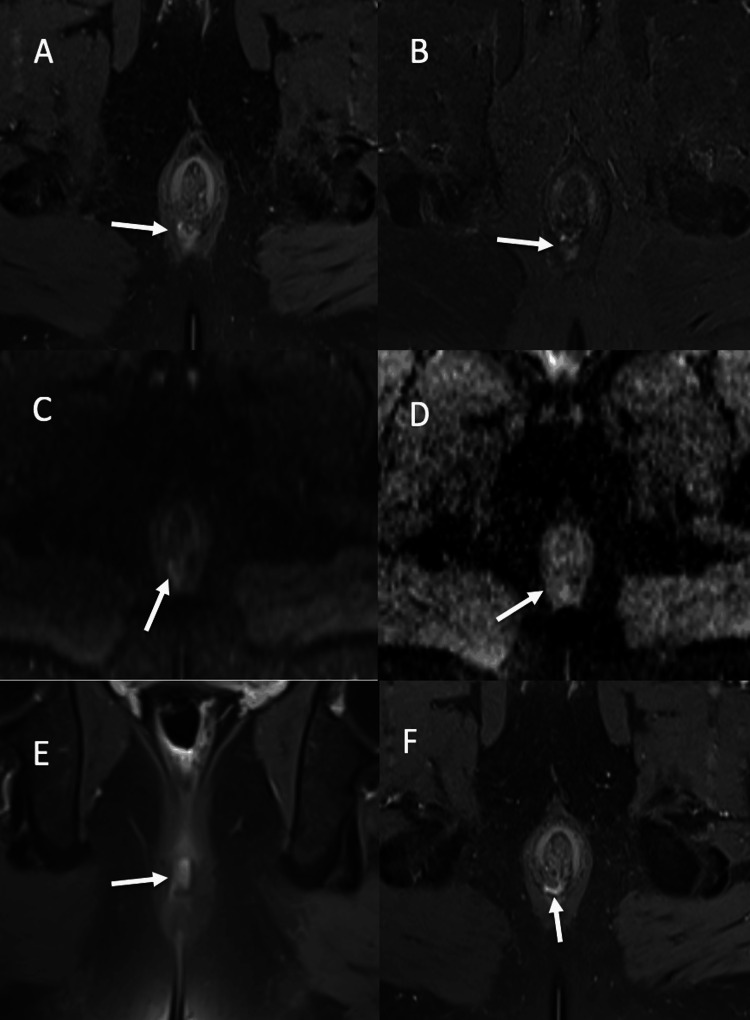
Perianal abscess in grade 2 intersphincteric fistula: (A-B) Axial post contrast fat suppressed T1-WI and axial STIR respectively, depicting a small abscess near internal opening of intersphincteric fistula showing heterogeneous enhancement on post contrast fat suppressed T1-WI (A) and hyperintense signal on STIR (B). (C-D) Axial DWI and ADC respectively, depicting abscess showing hyperintense signal on DWI (C) with corresponding signal drop on ADC (D). (E-F) Coronal and axial post contrast fat suppressed T1-WI respectively, showing intersphincteric fistula with internal opening at 6-o’clock position. T1-WI: T1 weighted image; STIR: short tau inversion recovery; DWI: diffusion weighted imaging, ADC: apparent diffusion coefficient

Figures 5-7 depict a few cases from our study to show the abscess characteristics by different MRI sequences in complicated grade 2 and 4 perianal fistula.

## Conclusions

Our study concluded that DWI is a very sensitive sequence with near equivalent accuracy to contrast-enhanced MRI in detection of perianal abscess in complicated fistulas. Although T2 STIR is also a sensitive sequence for identification of perianal abscess, DWI is superior to T2 STIR in differentiating perianal abscess from perianal inflammation with no abscess. As our study included abscesses in complicated fistula cases, we also found DWI to be better than T2 STIR in differentiating between active fistulas from non-specific perianal inflammation. Thus, for perianal abscess due to any cause, DWI can be used as an alternative to post contrast fat-suppressed MRI in accurately diagnosing and characterizing perianal abscess and differentiating it from surrounding non-specific inflammation.
